# Systemic Administration of CpG Oligodeoxynucleotide and Levamisole as Adjuvants for Gene-Gun-Delivered Antitumor DNA Vaccines

**DOI:** 10.1155/2011/176759

**Published:** 2011-10-18

**Authors:** Michal Šmahel, Ingrid Poláková, Eva Sobotková, Eva Vajdová

**Affiliations:** Department of Experimental Virology, Institute of Hematology and Blood Transfusion, U Nemocnice 1, 128 20 Prague 2, Czech Republic

## Abstract

DNA vaccines showed great promise in preclinical models of infectious and malignant diseases, but their potency was insufficient in clinical trials and is needed to be improved. In this study, we tested systemic administration of two conventional adjuvants, synthetic oligodeoxynucleotide carrying immunostimulatory CpG motifs (CpG-ODN) and levamisole (LMS), and evaluated their effect on immune reactions induced by DNA vaccines delivered by a gene gun. DNA vaccination was directed either against the E7 oncoprotein of human papillomavirus type 16 or against the BCR-ABL1 oncoprotein characteristic for chronic myeloid leukemia. High doses of both adjuvants reduced activation of mouse splenic CD8^+^ T lymphocytes, but the overall antitumor effect was enhanced in both tumor models. High-dose CpG-ODN exhibited a superior adjuvant effect in comparison with any combination of CpG-ODN with LMS. In summary, our results demonstrate the benefit of combined therapy with gene-gun-delivered antitumor DNA vaccines and systemic administration of CpG-ODN or LMS.

## 1. Introduction

After pioneering studies showing the expression of protein antigens from plasmid DNA and the ability of these antigens to induce both humoral and cell-mediated immunity in the early 1990s [1–3], DNA vaccines against some infectious diseases and also malignant tumors were quickly developed and successfully tested in animal models. However, the efficacy of DNA immunization in initial clinical trials was disappointing [4]. 

Immune reactions induced by DNA vaccines can be enhanced by adjuvants that are classified into two groups by Sasaki et al. [5]—genetic and conventional. While genetic adjuvants are plasmids producing cytokines, chemokines or other immunomodulatory molecules, conventional adjuvants are chemical compounds increasing or modulating immune responses. As genetic adjuvants are of the same nature as DNA vaccines, they can be easily codelivered in any method of DNA vaccine administration. However, conventional adjuvants can be mixed and codelivered with DNA vaccines injected as a solution, but their codelivery with DNA vaccines administered via a gene gun is limited by the mode of application. Only local application of the conventional adjuvant imiquimod was more widely tested in combination with gene-gun delivery of plasmid DNA [6, 7].

Of five conventional (chemical) adjuvants tested after addition to an intramuscular DNA vaccine, levamisole (LMS), a synthetic phenylimidazolthiazole, induced the strongest Th1 immune reactions [8]. The high immunostimulatory activity of LMS in DNA vaccination was confirmed in subsequent studies [9, 10]. This compound developed as an anthelmintic drug in the 1960s is also recommended, in combination with 5-fluorouracil, as adjuvant chemotherapy for colon cancer [11, 12].

Moreover, the effect of DNA vaccination is supported by immunostimulatory unmethylated CpG motifs that can be either carried by an immunization plasmid itself or delivered on synthetic oligodeoxynucleotides (ODNs) [13]. Demonstrated in animal models, the benefit of ODNs carrying CpG motifs (CpG-ODN) after addition to various types of vaccines was evaluated in clinical trials [14, 15]. However, systemic administration of ODNs caused suppression of splenic cytotoxic T lymphocytes (CTLs) in mice, which raised concerns for the usability of CpG-ODN in antitumor therapy. This effect was associated with enhanced production of indoleamine 2,3-dioxygenase (IDO) by splenic CD19^+^ dendritic cells (DCs) [16, 17]. Systemic injection of CpG-ODN also diminished cross-presentation of antigens by DCs [18]. On the other hand, repeated systemic administration of high doses of CpG-ODN induced immune-mediated protection from acute lymphoblastic leukemia [19].

In this study, we evaluated the influence of systemic administration of LMS and CpG-ODN on the activation of mouse splenic CTLs by gene-gun DNA vaccination and on the antitumor effect elicited in models of chronic myeloid leukemia (CML) and human-papillomavirus- (HPV-) induced tumors. These adjuvants were compared for potency and combined treatment was examined as well.

## 2. Materials and Methods

### 2.1. Plasmids

The plasmids pBSC [20], pBSC/bcr-abl [21], pBSC/E7GGG.GUS [22], and pBSC/EGGG.LAMP [23] were used for immunization. The plasmid pBSC/bcr-abl produces the protein BCR-ABL1 (p210) from the fusion gene generated by the b3a2 chromosomal translocation t(9; 22) in a CML patient. The fusion gene E7GGG.GUS consists of the mutated HPV16 E7 gene (E7GGG) containing three point mutations resulting in substitutions D21G, C24G, and E26G in the Rb-binding site [20] and the gene encoding *E. coli* *β*-glucuronidase (GUS). In the E7GGG.LAMP gene, E7GGG was fused with two signal sequences of lysosome-associated membrane protein 1 (LAMP-1).

### 2.2. Cell Lines

TC-1 cells, kindly provided by T. C. Wu (Johns Hopkins University, Baltimore, Md), were prepared by the transformation of C57BL/6 mouse primary lung cells with the HPV16 E6/E7 oncogenes and the activated human *H-ras* gene [24]. TC-1 cells were grown in high glucose Dulbecco's Modified Eagle's Medium (DMEM; PAA Laboratories, Linz, Austria) supplemented with 10% fetal calf serum (FCS; PAA), 2 mM L-glutamine, 100 U/mL penicillin, and 100 *μ*g/mL streptomycin.

12B1 cells producing the BCR-ABL1 (b3a2) protein [25] were obtained through the courtesy of E. Katsanis (University of Arizona, Tucson, Ariz). They were derived by transformation of BALB/c mouse primary bone marrow cells with a retrovirus-derived vector carrying the BCR-ABL1 fusion gene. 12B1 cells were passaged in RPMI-1640 medium (Sigma-Aldrich, St. Louis, Mont) supplemented with 10% FCS, 1 mM pyruvate, 50 mM 2-mercaptoethanol, L-glutamine, and antibiotics. 

### 2.3. Mice

Six- to eight-week-old female C57BL/6 (H-2^b^) or BALB/c mice (H-2^d^; Charles River, Germany) were used in immunization experiments. Animals were maintained under standard conditions at the Center for Experimental Biomodels, Charles University, Prague.

### 2.4. Immunization Experiments

Plasmid DNA was coated onto 1 *μ*m gold particles (Bio-Rad, Hercules, Calif) as described previously [20]. Mice were immunized with plasmids by a gene gun (Bio-Rad) at a discharge pressure of 400 psi into the shaven skin of the abdomen. Each immunization consisted of one or two shots delivering 1 or 2 *μ*g of plasmid DNA.

For *in vitro* examination of immune reactions, C57BL/6 mice (three per group) were immunized with two 1 *μ*g doses of the E7GGG.GUS plasmid given one week apart. In therapeutic immunization experiments, C57BL/6 or BALB/c mice (six per group) were first s.c. administered 3 × 10^4^ TC-1 or 5 × 10^3^ 12B1 cells suspended in 150 *μ*L or 200 *μ*L PBS, respectively, into the back and then vaccinated with pBSC/E7GGG.LAMP (1 *μ*g doses three and ten days after cell inoculation) or pBSC/bcr-abl (2 *μ*g doses three, six, and ten days after cell inoculation), respectively. The empty pBSC plasmid was used as a negative control. Tumor cells were administered under anesthesia with intraperitoneal etomidate (0.5 mg/mouse; Janssen Pharmaceutica, Beerse, Belgium). Tumor growth was monitored twice a week, and tumor size was calculated from three perpendicular measurements using the formula (*π*/6) (a × b × c). Mice were sacrificed when tumor volume reached 1 cm^3^ or two months after cell inoculation.

The adjuvants phosphorothioate-stabilized oligodeoxynucleotide ODN1826 carrying CpG immunostimulatory motifs (TCCATGACGTTCCTGACGTT; Generi Biotech, Hradec Kralove, Czech Republic) and LMS (Sigma-Aldrich) dissolved in 200 *μ*L PBS were i.p. injected on the days of DNA vaccination.

### 2.5. Tetramer Staining

A week after immunization with pBSC/E7GGG.GUS, tetramer staining was performed as described previously [26]. In brief, lymphocyte bulk cultures were prepared from splenocytes of three immunized animals and restimulated with the HPV16 E7_49-57_ peptide (RAHYNIVTF) for 6 days. After incubation with anti-mouse CD16/CD32 antibody (Fc-block; BD Biosciences, San Diego, Calif), lymphocytes were stained with a mixture of H-2D^b^/E7_49-57_-PE tetramers (Sanquin, Amsterdam, The Netherlands) and anti-mouse CD8a-FITC antibody (BD Biosciences). The stained cells were measured on a Coulter Epics XL flow cytometer (Coulter, Miami, Fla) and analyzed by FlowJo 7.2.2 software (TreeStar, Ashland, Ore).

### 2.6. Statistical Analysis

Tumor growth was evaluated by two-way analysis of variance, tumor formation by log-rank test, and the expansion of E7-specific splenocytes in tetramer assay by Student's *t* test. Results were considered significantly different if *P* < 0.05. Calculations were performed using GraphPad Prism 5.0 software (GraphPad Software, San Diego, Calif).

## 3. Results

### 3.1. Systemic Administration of High-Dose CpG-ODN or LMS Supports Antitumor Effect of Gene-Gun DNA Vaccination

We tested the influence of systemic application of CpG or LMS on antitumor effect induced by DNA vaccines delivered with a gene gun in two mouse tumor models: TC-1 cells producing the HPV16 E7 oncoprotein and 12B1 cells producing the BCR-ABL1 fusion protein injected s.c. into C57BL/6 and BALB/c mice, respectively. Because of high efficacy of immunization against the E7 antigen, we used the pBSC/E7GGG.LAMP plasmid that is less immunogenic than pBSC/E7GGG.GUS and applied only two 1 *μ*g doses. The plasmid pBSC/bcr-abl is potent in preventive immunization against 12B1 cells [27], but its efficacy in therapeutic immunization is low. Therefore, vaccination against 12B1 cells consisted of three 2 *μ*g doses.

**Figure 1:**
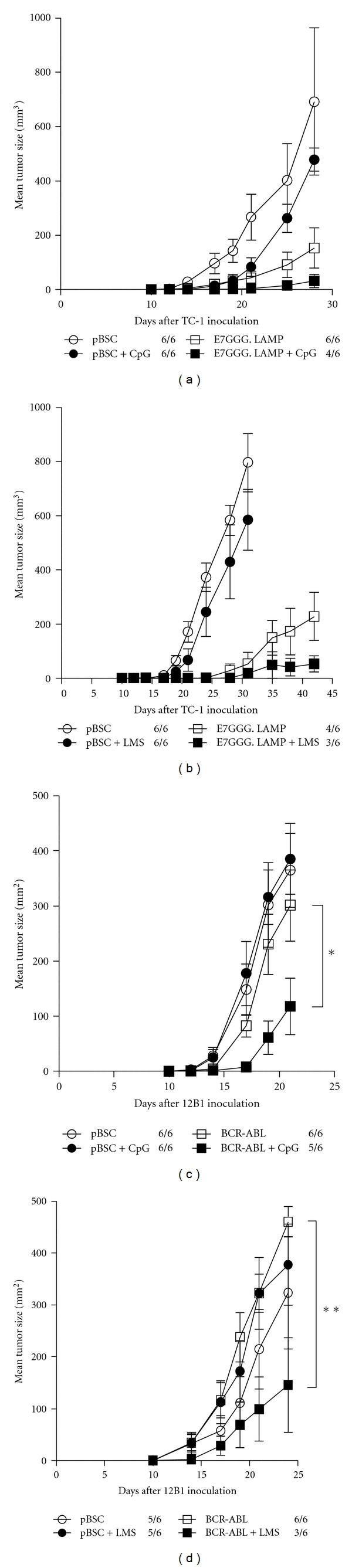
Antitumor effect of systemic administration of high-dose adjuvants. C57BL/6 or BALB/c mice (*n* = 6) were s.c. inoculated with 3 × 10^4^ TC-1 (a, b) or 5 × 10^3^ 12B1 cells (c, d) and immunized by a gene gun with 1 *μ*g of pBSC/E7GGG.LAMP three and ten days later or with 2 *μ*g of pBSC/bcr-abl three, six, and ten days later, respectively. The pBSC plasmid was used as a negative control. CpG-ODN (50 *μ*g; a, c) or LMS (200 *μ*g; b, d) was i.p. injected on the days of DNA vaccination. No. of mice with a tumor/no. of mice in the group is indicated. Bars: ±SD; *P < 0.05; **P < 0.01.

For initial experiments, we chose relatively high doses of adjuvants: 50 *μ*g of CpG-ODN and 200 *μ*g of LMS. Both adjuvants reduced the growth of TC-1-induced tumors in animals either immunized or nonimmunized against the E7 antigen, but this effect was nonsignificant (Figures 1(a) and 1(b)). However, while adjuvants alone did not affect 12B1-induced tumors, they significantly reduced tumor growth after combination with vaccination (CpG-ODN: *P* = 0.027, LMS: *P* = 0.008; Figures 1(c) and 1(d)). Moreover, in both tumor models, administration of adjuvants to immunized mice resulted in inhibition of tumor formation in a portion of animals. This effect was significant for combination of pBSC/bcr-abl immunization and LMS administration (*P* = 0.019).

### 3.2. Systemic Administration of High Doses of CpG-ODN or LMS Reduces the Stimulation of Splenic CTLs by Gene-Gun DNA Vaccination

**Figure 2:**
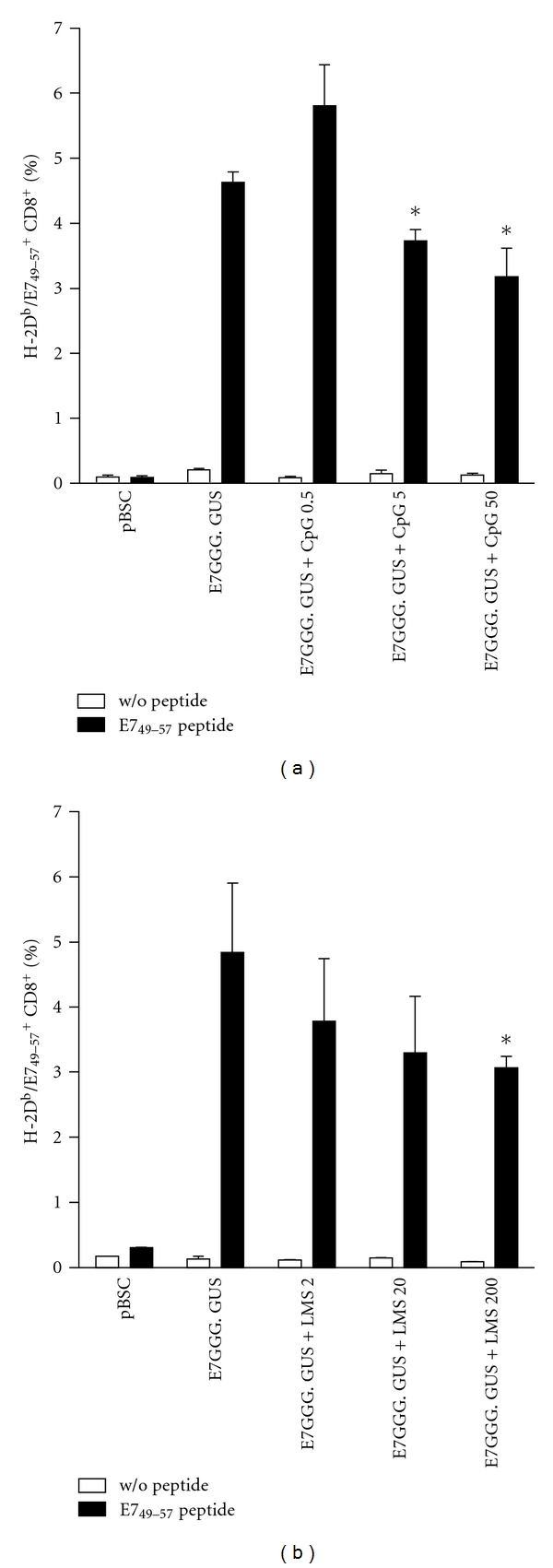
Detection of E7-specific splenic CTLs after DNA vaccination combined with systemic administration of adjuvants. C57BL/6 mice (*n* = 3) were twice immunized at a 1-week interval with 1 *μ*g of pBSC or pBSC/E7GGG. GUS by a gene gun and i.p. injected with indicated doses of CpG-ODN (a) or LMS (b). One week after the second immunization, lymphocyte bulk cultures were prepared from splenocytes, restimulated with the RAHYNIVTF peptide for 6 days, and stained with a mixture of H-2D^b^/E7_49-57_-PE tetramers and anti-mouse CD8a-FITC antibody. Control lymphocytes were cultured without the peptide. Columns: mean of duplicate samples; bars: ±SD; *P < 0.05 (the comparison with the E7GGG.GUS group).

As systemic inoculation of 50 *μ*g of CpG-ODN has been reported to reduce the CTL activity induced by immunization [16, 17], we evaluated this effect for CpG-ODN and LMS after vaccination of C57BL/6 mice with the pBSC/E7GGG.GUS plasmid. The stimulation of splenic CTLs specific for the H-2D^b^ E7 epitope was measured after the addition of CpG-ODN or LMS at doses used for enhancement of the antitumor effect of the DNA vaccines (i.e., 50 *μ*g and 200 *μ*g, resp.) and two lower doses (5 and 0.5 *μ*g for CpG-ODN and 20 and 2 *μ*g for LMS). Both adjuvants exhibited reduction of E7-specific CD8^+^ T lymphocytes in the spleens (Figure 2). The extent of this inhibition was similar for the highest (*P* < 0.05) and medium doses of CpG-ODN and LMS, but, while the lowest dose of LMS was still inhibiting CTL response (Figure 2(b)), that of CpG-ODN moderately increased it (Figure 2(a)).

### 3.3. Combination of CpG-ODN and LMS Does Not Outperform CpG-ODN Alone in Supporting Antitumor Effect of Gene-Gun DNA Vaccination

**Figure 3:**
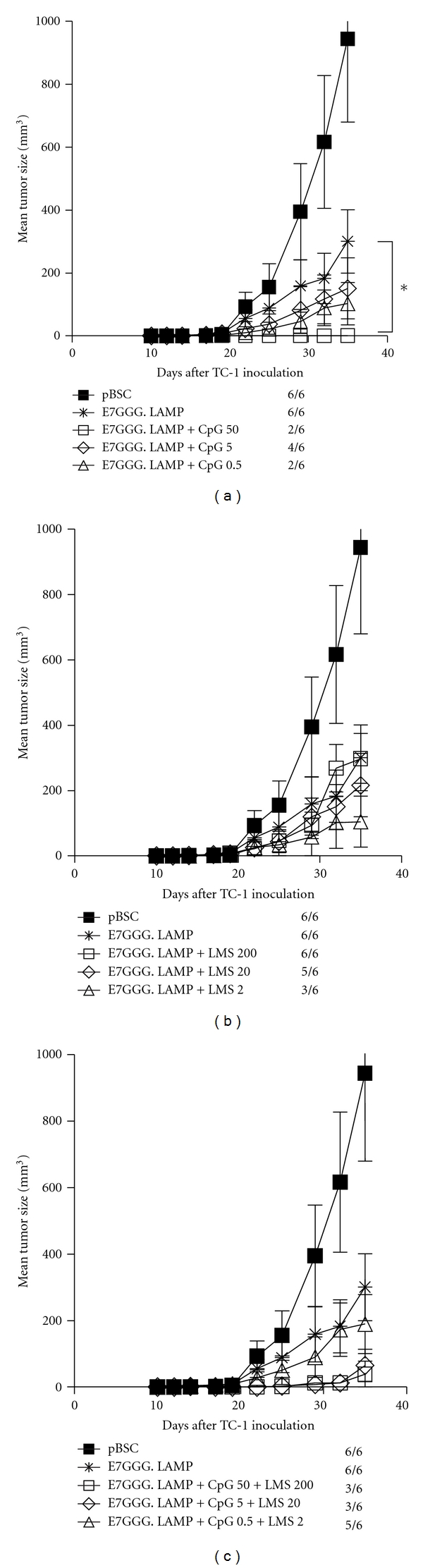
Antitumor effect of systemic administration of single adjuvants or their combinations. C57BL/6 mice (*n* = 6) were s.c. inoculated with 3 × 10^4^ TC-1 cells and immunized by a gene gun with 1 *μ*g of pBSC/E7GGG.LAMP three and ten days later. The pBSC plasmid was used as a negative control. CpG-ODN (a), LMS (b), or their combinations (c) were i.p. injected at indicated doses on the days of DNA vaccination. The graphs (a), (b), and (c) were constructed from the results of the same experiment. No. of mice with a tumor/no. of mice in the group is indicated. Bars: ±SD; *P < 0.05.

Showing lower inhibition of CTLs with lower doses of adjuvants, we next compared the antitumor effects of the three doses in the model of TC-1-induced tumors. Simultaneously, we tested combinations of both adjuvants. In control mice immunized with the pBSC/E7GGG.LAMP plasmid, tumor growth was markedly reduced in comparison with pBSC-treated mice, but tumors developed in all animals (Figure 3). For CpG-ODN, the highest dose of the adjuvant (50 *μ*g) most efficiently supported antitumor immunity elicited by DNA vaccination—tumor formation was inhibited in four out of six mice (*P* = 0.005) and tumor growth was significantly reduced (*P* = 0.033; Figure 3(a)). Conversely, the administration of the lowest dose of LMS (2 *μ*g) resulted in the lowest tumor rate (3/6, *P* = 0.034; Figure 3(b)). Combinations of high and medium doses of adjuvants provided the best antitumor effects (tumor rate 3/6, *P* = 0.020 and *P* = 0.041, resp.; Figure 3(c)), but none of them outperformed the high-dose CpG-ODN in terms of potency.

## 4. Discussion

Successful examination of DNA vaccines in animals resulted in license acquisition by several veterinary vaccines directed against both infectious and malignant diseases. The evaluation of DNA vaccines in clinical trials showed that these vaccines were well tolerated and safe, but their immunogenicity was unexpectedly lower than in preclinical models. In recent years, progress in enhancing the efficacy of DNA vaccination in humans has been achieved mainly thanks to the improvement of physical delivery methods, with muscle electroporation and particle bombardment of the skin being currently predominant [28]. 

Utilization of adjuvants that is crucial for a high efficacy of protein and peptide vaccines is still in its infancy in DNA immunization. Their introduction into clinical immunization with DNA vaccines could be another step in the enhancement of DNA vaccination efficacy. In this study, we tested systemic administration of two adjuvants, CpG-ODN and LMS, in combination with gene-gun DNA immunization and evaluated adjuvant-mediated impact on the antitumor effect induced by DNA vaccines. 

At high doses, both adjuvants reduced activation of specific splenic CTLs, but, overall, they enhanced the antitumor potency of DNA vaccination. Inhibition of splenic CTLs by CpG-ODN has already been reported, and increased expression of IDO by splenic CD19^+^ DCs has been identified as a key factor in this process [16, 17]. However, CpG-ODN directly or indirectly affects other immune cells, including different types of DCs, T cells, NK cells, B cells, monocytes, and neutrophils, that can contribute to reduced tumor growth [29]. Similarly, LMS activates DCs and induces their maturation, which leads to stimulation of CTLs [30]. Thus, complex activation of the immune system by the two systemically delivered adjuvants can result in strengthened immunity in the tumor despite mild immunosuppression in the spleen.

CpG-ODN and LMS activate DCs by binding to Toll-like receptor- (TLR-) 9 and TLR-2 [30], respectively. Both adjuvants induce production of IL-12 and stimulate Th1 immune response. Our comparison showed higher potency of CpG-ODN in enhancement of antitumor effect against mouse TC-1 tumor cells. Combinations of various doses of CpG-ODN and LMS did not further increase the impact on tumor growth. However, subsets of mouse and human DCs differ in TLR-9 and TLR-2 expression [31]: while all mouse DC subsets produce both TLRs, human myeloid DCs produce only TLR-2 and plasmacytoid DCs only TLR-9. Then, in humans, the combination of CpG-ODN and LMS can be useful in antitumor treatment.

Systemic administration of CpG-ODN is well tolerated and induces Th1 immune response in humans [32]. As preclinical models demonstrated improved effect of chemotherapy after addition of CpG-ODN, clinical trials examining this combined treatment have also been launched [14]. Furthermore, recent results in mouse tumor models suggested the potential of systemic administration of CpG-ODN in the inhibition of metastasis [33] and treatment of minimal residual disease [19]. This study showed that vaccination could supplement such methods of antitumor therapy with systemic CpG-ODN delivery.

## 5. Conclusions

Our results demonstrate that in spite of partial inhibition of specific immunity by systemic administration of high-dose CpG-ODN or LMS, these adjuvants potentiated the antitumor effect of DNA vaccines delivered by a gene gun. CpG-ODN was more efficient than LMS, and combination of both adjuvants did not outperform CpG-ODN alone in terms of potency. To conclude, we propose a new approach to enhancing antitumor gene-gun DNA vaccination: systemic CpG-ODN delivery.
